# Cell wall dynamics modulate acetic acid-induced apoptotic cell death of *Saccharomyces cerevisiae*

**DOI:** 10.15698/mic2014.09.164

**Published:** 2014-08-27

**Authors:** António Rego, Ana M. Duarte, Flávio Azevedo, Maria J. Sousa, Manuela Côrte-Real, Susana R. Chaves

**Affiliations:** 1 Centro de Biologia Molecular e Ambiental, Departamento de Biologia, Universidade do Minho, Braga, Portugal.

**Keywords:** yeast, apoptosis, acetic acid, MAPK, CWI

## Abstract

Acetic acid triggers apoptotic cell death in *Saccharomyces cerevisiae*, similar to mammalian apoptosis. To uncover novel regulators of this process, we analyzed whether impairing MAPK signaling affected acetic acid-induced apoptosis and found the mating-pheromone response and, especially, the cell wall integrity pathways were the major mediators, especially the latter, which we characterized further. Screening downstream effectors of this pathway, namely targets of the transcription factor Rlm1p, highlighted decreased cell wall remodeling as particularly important for acetic acid resistance. Modulation of cell surface dynamics therefore emerges as a powerful strategy to increase acetic acid resistance, with potential application in industrial fermentations using yeast, and in biomedicine to exploit the higher sensitivity of colorectal carcinoma cells to apoptosis induced by acetate produced by intestinal propionibacteria.

## INTRODUCTION

*Saccharomyces cerevisiae* is currently a well-established eukaryotic model organism used in the elucidation of molecular mechanisms of programmed cell death (PCD) pathways 1. In particular, acetic acid-induced apoptosis is among the best-characterized yeast apoptotic pathways, due to the interest of modulating this response for applications in both biotechnology and biomedicine 2. Indeed, there is an increasing number of studies aiming to develop improved yeast strains for use in fermentations, a process often hindered by excessive levels of acetic acid 3,4. On the other hand, it has been found that colorectal carcinoma (CRC) cells are particularly sensitive to short-chain fatty acids produced by propionibacteria (including acetate) that reside in the intestine, generating an interest in exploring novel probiotics as a prevention/therapeutic tool in CRC 5,6,7,8. In yeast, acetic acid triggers a PCD process with features similar to mammalian apoptosis, such as exposure of phosphatidylserine on the outer leaflet of the cytoplasmic membrane, chromatin condensation and DNA fragmentation 9. Like in mammalian cells, mitochondria play a key role in this process. Indeed, different alterations in mitochondrial structure and function occur during acetic acid-induced apoptosis, including reduction in cristae number and mitochondrial swelling 10, a transient mitochondrial hyper-polarization followed by depolarization, production of reactive oxygen species (ROS), decrease in cytochrome oxidase activity and mitochondrial outer membrane permeabilization (MOMP), with concomitant release of cytochrome *c* and yeast Aif1p 11,12,13. Several proteins regulating acetic acid-induced apoptosis have already been identified, such as Por1p (yeast voltage dependent anion channel), which protects cells from apoptosis triggered by acetic acid, and ADP/ATP carrier proteins, yeast orthologs of the adenine nucleotide transporter, which seem to mediate MOMP and cytochrome *c* release 14. Mitochondrial proteins involved in fission/fusion, namely Fis1p, Dnm1p and Mdv1p 15, have also been implicated in the execution of the yeast apoptotic program induced by acetic acid, as has the cathepsin D homologue Pep4p, important for mitochondrial degradation in this process 16. The Ras-cAMP-PKA pathway has also been shown to mediate acetic acid-induced apoptosis, both in *S. cerevisiae* and *C. albicans* 17. Despite the large number of proteins shown to be involved, the complexity of the networks contributing to acetic acid-induced cell death and their interrelationships are still elusive.

Mitogen Activated Protein Kinase (MAPK) cascades are important signaling pathways that allow yeast cells to adjust to changing environment conditions. These pathways regulate various important processes, from cell proliferation and differentiation to cell death. MAPK cascades normally contain three protein kinases that act in sequence: a MAP kinase kinase kinase (MAPKKK, MAP3K, MEKK or MKKK), a MAP kinase kinase (MAPKK, MAP2K, MEK or MKK), and a MAP kinase (MAPK). Therefore, when the cascade is activated, the MAPKKK phosphorylates the MAPKK, which in turn phosphorylates both the threonine and tyrosine residues of a conserved -Thr-X-Tyr- motif within the activation loop of the MAPK 18. MAPKs phosphorylate a diverse set of well-characterized substrates, including transcription factors, translational regulators, MAPK-activated protein kinases (MAPKAP kinases), phosphatases, and other classes of proteins, thereby regulating metabolism, cellular morphology, cell cycle progression, and gene expression in response to a variety of extracellular stresses and molecular signals 19. The specificity of the MAPK pathways is regulated at several levels, including kinase-kinase and kinase-substrate interactions, co-localization of kinases by scaffold proteins, and inhibition of cross-talk/output by the MAPKs themselves 20. *S. cerevisiae* contains five MAPKs, Fus3p, Kss1p, Hog1p, Slt2p/Mpk1p and Smk1p, in five functionally distinct cascades, associated with the mating-pheromone response, invasive growth/pseudohyphal development, high osmolarity, cell wall integrity (CWI), and sporulation, respectively 21. The five MAP kinases are controlled by four MAPKKs, Ste7p (regulating Fus3p and Kss1p), Pbs2p (regulating Hog1p) and the redundant pair Mkk1p/Mkk2p (regulating Slt2p/Mpk1p), and by four MAPKKKs, Ste11p, the redundant pair Skk2p/Skk22p and Bck1p. The specificity of signal transduction is guaranteed by scaffold proteins 22, Ste5p for the mating-pheromone response pathway, and Pbs2p for the High Osmolarity Glycerol (HOG) pathway.

It has been reported that exposure to non-lethal concentrations of acetic acid activates the HOG pathway 23, and also leads to phosphorylation of Slt2p, a MAPKK from the CWI pathway 24. These results suggest an intricate relation between CWI and HOG signaling in response to growth in the presence of acetic acid. In this work, we aimed to characterize the involvement of MAPK signaling pathways in cell death induced by acetic acid in *S. cerevisiae*.

**Figure 1:**
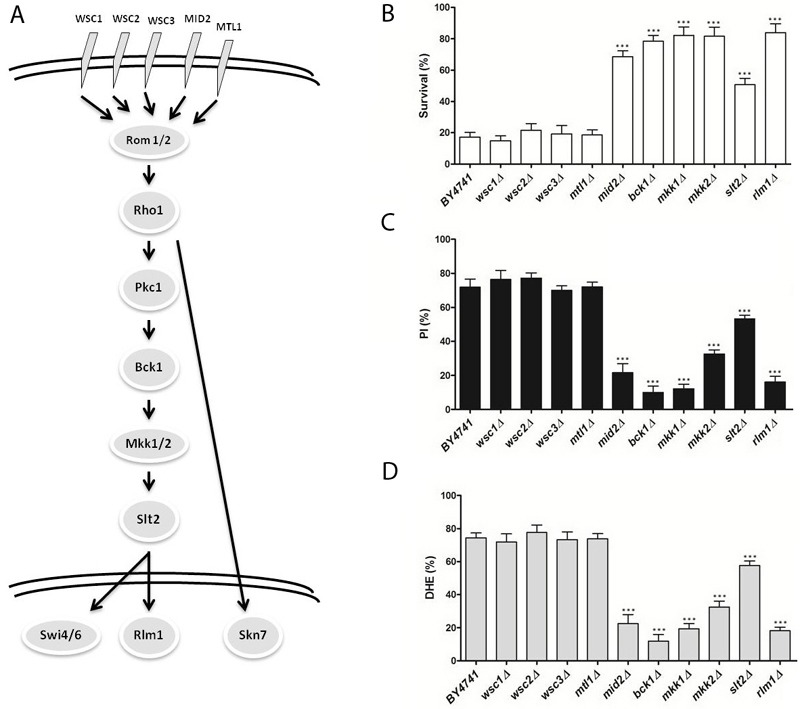
FIGURE 1: The role of the cell wall integrity (CWI) signaling pathway in acetic acid-induced apoptosis. **(A)** Overview of the pathway. **(B)** Survival of the wild type (BY4741) and indicated isogenic yeast strains exposed to 110 mM acetic acid, at pH 3.0 for 200 min. Values represent means ± SD of at least three independent experiments. **(C)** Percentage of cells displaying propidium iodide (PI) internalization assessed by flow cytometry after treatment with 110 mM acetic acid, at pH 3.0 for 200 min. **(D)** Percentage of intracellular ROS levels assessed by flow cytometry after treatment with 110 mM acetic acid, at pH 3.0 for 200 min. Values in (C) and (D) are represented as means ± SD of at least three independent experiments with at least 20000 cells counted in each time point. Asterisks represent significant statistical difference from control by One-way ANOVA test: (* represents p < 0.05 and *** p < 0.001).

## RESULTS

### Components of the MAPK pathways modulate acetic acid-induced cell death

**Figure 2:**
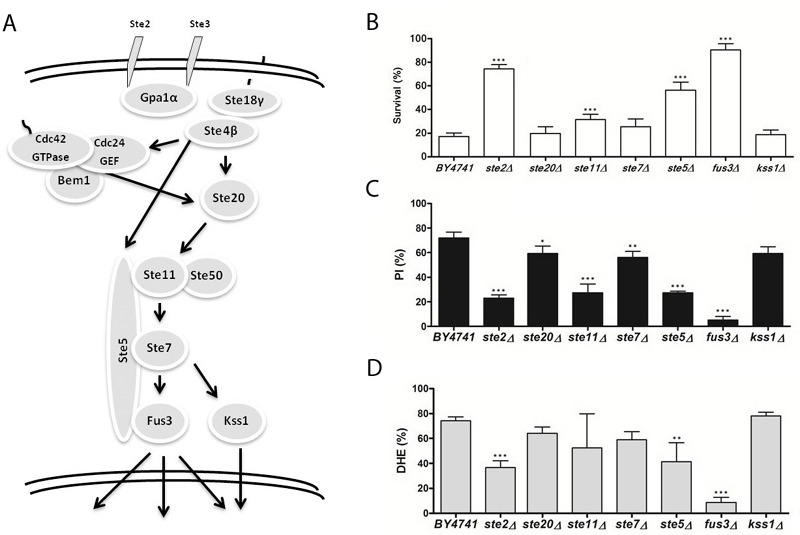
FIGURE 2: The role of the mating-pheromone response signaling pathway in acetic acid-induced apoptosis. (A-C) panels as described in Figure 1.

In order to investigate the involvement of the different MAPK signaling pathways in acetic acid-induced cell death, we assessed whether deletion of components of these pathways affected the viability of *S. cerevisiae* cells in response to acetic acid. In Figures 1 through 4, a simplified model of the MAPK pathways is represented in the (A) panels, and the viability of the different mutants is shown in the (B) panels. We found that several mutants of the MAPK components were significantly more resistant to acetic acid-induced cell death than the wild type strain. These included multiple components of the mating-pheromone response pathway (*ste2*Δ, s*te5*Δ and *fus3*Δ) and most components of the cell wall integrity pathway (mutants *mid2*Δ, *bck1*Δ, *mkk1*Δ/*mkk2*Δ, *slt2/mpk1*Δ). The mutants *sho1*Δ and *msb2*Δ, lacking the two membrane signaling proteins common to both invasive growth/pseudohyphal development and of the HOG pathway, and the mutant *ssk22*Δ, a member of the redundant pair of MAPKKK of the HOG pathway, were also significantly more resistant to acetic acid-induced cell death than the wild type strain.

**Figure 3:**
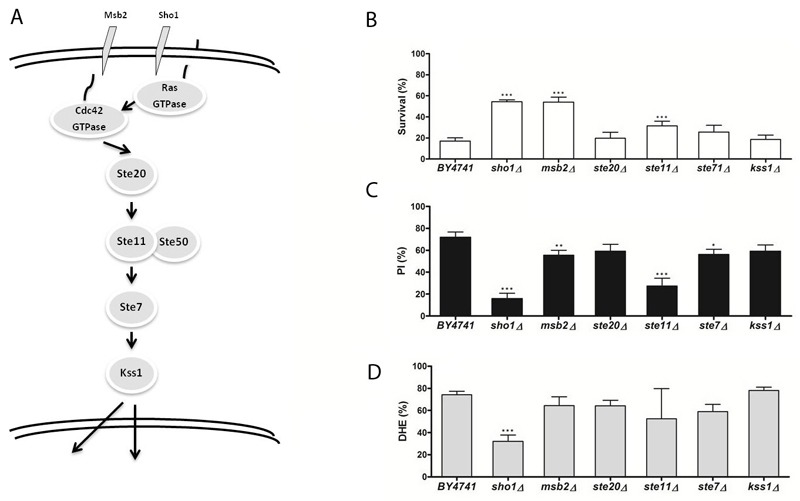
FIGURE 3: The role of the invasive growth/pseudohyphal development signaling pathway in acetic acid-induced apoptosis. (A-C) panels as described in Figure 1.

**Figure 4:**
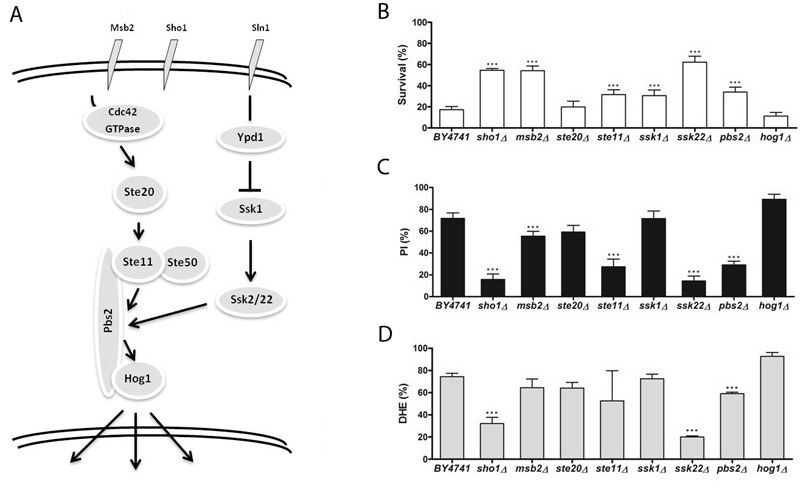
FIGURE 4: The role of the high osmolarity glycerol (HOG) signaling pathway in acetic acid-induced apoptosis. (A-C) panels as described in Figure 1.

Acetic acid induces a mitochondria-dependent apoptotic cell death in *S. cerevisiae* that displays characteristic apoptotic markers such as ROS accumulation, phosphatidylserine externalization, chromatin condensation, DNA fragmentation and mitochondrial dysfunction with release of cytochrome *c* 9,12. We therefore also assessed loss of plasma membrane integrity and ROS accumulation in the mutant strains exposed to acetic acid by staining cells with PI and DHE, respectively, and analyzing the fluorescence by flow cytometry. We found that, in general, mutant strains with higher resistance to acetic acid had a lower percentage of cells displaying an accumulation of ROS and a lower percentage of cells with compromised plasma membrane integrity than the wild type strain (Fig. 1-4, C and D panels), confirming the involvement of the mating-pheromone response, HOG and CWI pathways, but not the invasive growth/pseudohyphal development pathway, in acetic acid-induced regulated cell death. In fact, though deletion mutants of some components of the latter pathway display a resistance phenotype, namely Msb2p, Sho1p and Ste11p, they are shared by other pathways, and the only MAPK of the pathway, Kss1p, does not seem to be involved.

**Figure 5:**
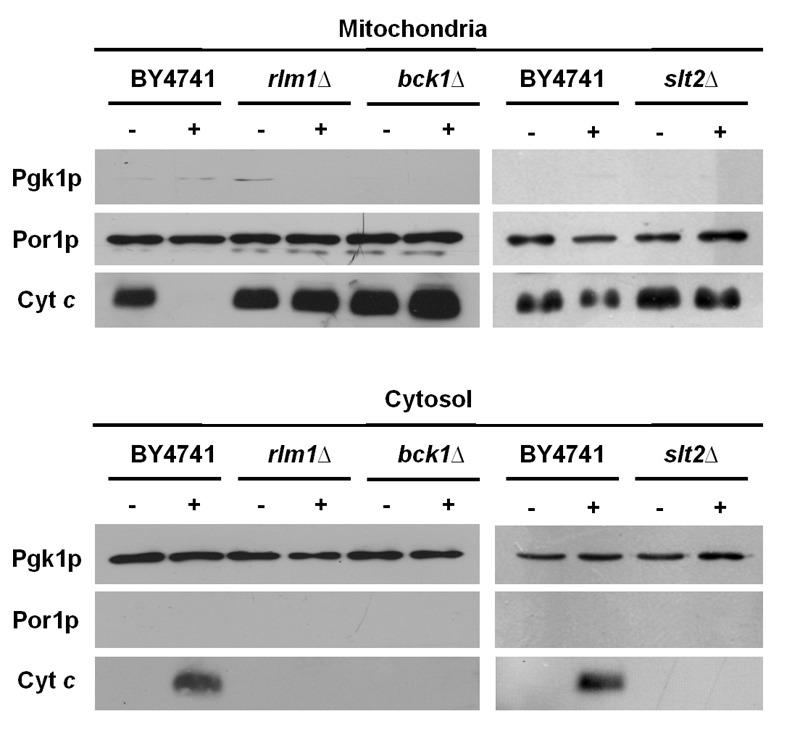
FIGURE 5: CWI mutants are defective in acetic acid-induced cytochrome *c* release. Western blot analysis of cytochrome *c* in *S. cerevisiae* strains BY4741, *bck1*Δ,* slt2*Δ and* rlm1*Δ before (-) and after (+) exposure to 120 mM acetic acid, pH 3.0, for 200 min, in both mitochondrial and cytosolic fractions. Cytosolic phosphoglycerate kinase (Pgk1p) and mitochondrial porin (Por1p) levels were used as loading control of cytosolic and mitochondrial fractions, respectively. A representative experiment is shown of at least two independent experiments with similar results.

As mentioned above, we have previously shown that acetic acid triggers a cell death program with hallmarks of mitochondria-dependent apoptosis, including MOMP and translocation of cytochrome *c* from the mitochondria into the cytosol. Since all mutants in the CWI MAPKKK/MAPKK/MAPK cascade were more resistant to acetic acid and displayed lower ROS accumulation, we next determined whether there was also decreased MOMP, to further support the involvement of mitochondria in the regulation of acetic acid-induced programmed cell death by the CWI signaling pathway. To this end, we assessed the levels of cytochrome *c* in cytosolic and mitochondrial extracts of untreated and acetic acid-treated cultures of wild type BY4741 and the CWI mutants *bck1*Δ and *slt2*Δ (deletion mutants of the MAPKKK and the MAPK of the pathway, respectively). In agreement with our previous results 12, exposure of wild type cells to acetic acid resulted in depletion of cytochrome *c* from mitochondria and consequent detection in the cytosolic fraction (Fig. 5). In contrast, we did not detect any depletion of cytochrome *c* from mitochondria or its translocation to the cytosol in acetic acid-treated *bck1*Δ or *slt2*Δ mutant cells, indicating that the CWI pathway mediates acetic acid-induced apoptosis through a mitochondrial pathway.

### Over-activation of the CWI pathway sensitizes cells to acetic acid exposure

**Figure 6:**
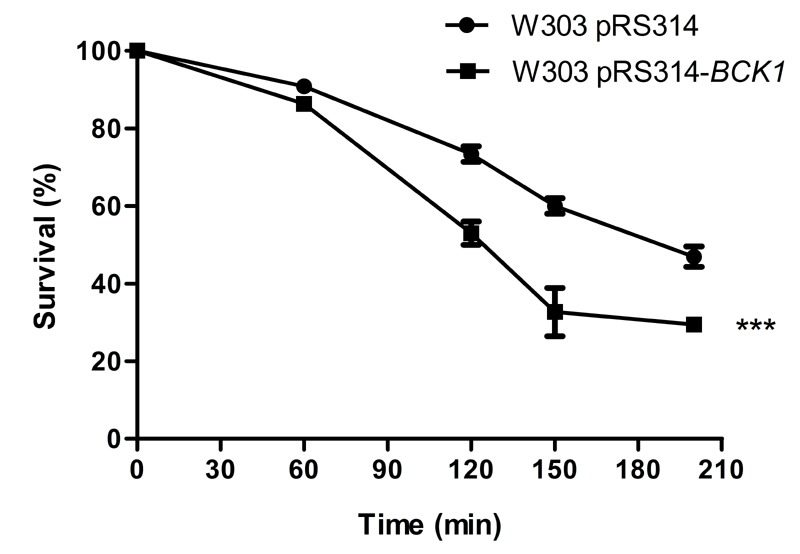
FIGURE 6: Stimulation of the CWI pathway sensitizes cells to acetic acid-induced cell death. Survival of wild type (W303) cells over-expressing Bck1p (pRS314-*BCK1-20*) or empty plasmid control (pRS314) exposed to 90 mM acetic acid, at pH 3.0 for 200 min. Values represent means ± SD of at least three independent experiments. Asterisks represent significant statistical difference from control by Two-way ANOVA test: (p < 0.001).

As shown above, impairment of the CWI pathway results in increased resistance to acetic acid-induced cell death. We therefore next sought to determine whether over-activation of this pathway would result in increased sensitivity to acetic acid. We transformed wild type cells with a plasmid expressing *BCK1-20* and respective empty plasmid control 25, as it has previously been shown that over-expression of this *BCK1* allele resulted in constitutive activation of the CWI pathway. We used the *S. cerevisiae *W303 strain as the wild type control due to the plasmid selective marker, and confirmed the *bck1*Δ mutant in this background also displayed resistance (not shown). Indeed, we found that over-expression of the Bck1 protein led to increased sensitivity to acetic acid (Fig. 6), providing further evidence that induction of the CWI pathway mediates acetic acid-induced cell death.

### CWI pathway mutants display differential sensitivity to multiple stresses

**Figure 7:**
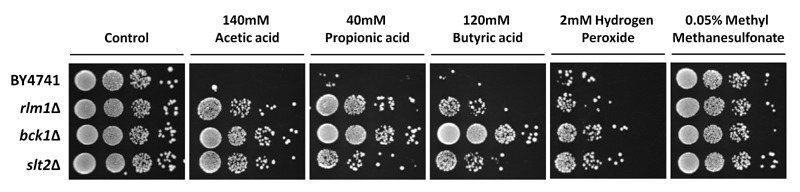
FIGURE 7: Sensitivity of CWI mutants to different stimuli. Survival of the wild type (BY4741) and indicated isogenic yeast strains exposed to 140 mM acetic acid, pH 3.0, 40 mM propionic acid, pH 3.0, 120 mM butyric acid, pH 3.0, 2 mM hydrogen peroxide, or 0.05% methyl methanesulfonate for 180 minutes at 30°C. Representative images are shown from at least 3 independent experiments.

To determine whether CWI mutants are specifically resistant to acetic acid-induced cell death or to death stimuli in general, we assessed the sensitivity of *bck1*Δ, *slt2*Δ, and *rlm1*Δ mutants to other cell death inducers by semi-quantitative spot assay (Fig. 7). All mutants were more resistant to acetic, propionic and butyric acid-induced cell death than the wild type strain, though to a different extent. Mutants were also slightly resistant to hydrogen peroxide-induced cell death, but not to methyl methanesulfonate-induced cell death. This indicates that the CWI pathway is particularly involved in acid-induced cell death, but is not a general stress response pathway.

### CWI pathway mutants are more sensitive to zymolyase digestion after acetic acid treatment

**Figure 8:**
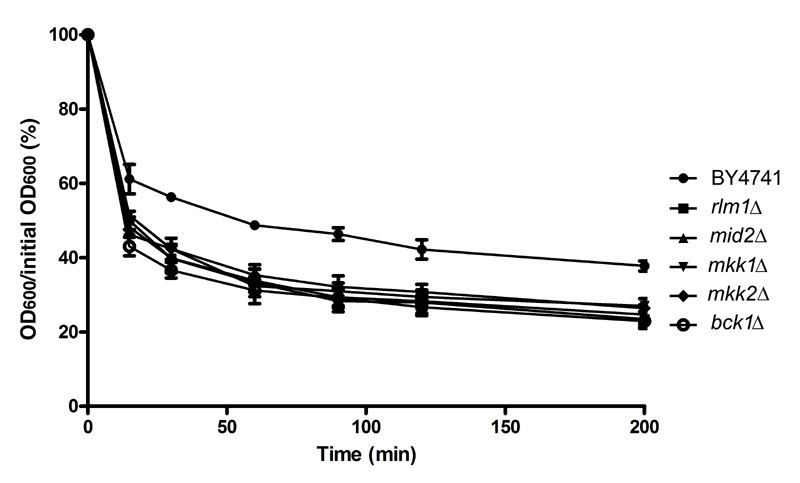
FIGURE 8: Sensitivity of CWI mutants to digestion with zymolyase. Cells were exposed to 110 mM acetic acid, at pH 3.0 for 200 min, digested with zymolyase 20T for up to 200 min, and optical density (600 nm) assessed over time. Values represent means ± SD of three independent experiments.

Mutants in which signaling through the upstream components or through the MAP kinase cascade of the CWI pathway is blocked display cell wall defects with varying degrees of severity and are more sensitive to a variety of stimuli 26. However, we determined that many of these mutants are more resistant to acetic acid-induced cell death. It has also been reported that weak-acid stress leads to cell wall remodeling, decreasing cell wall porosity 27. We therefore assessed whether there were differences in cell wall structural integrity of CWI mutants *mid2*Δ, *bck1*Δ, *mkk1*Δ and *mkk2*Δ in comparison with wild type cells after exposure to acetic acid, using a zymolyase sensitivity assay. All the CWI mutants tested were more susceptible to digestion with zymolyase after exposure to acetic acid than the wild type strain (Fig. 8), indicating that they display a resistant phenotype despite their cell wall defect.

### Rlm1p and its target genes involved in cell wall organization/biogenesis and cell wall structure modulate acetic acid-induced cell death

The final and most prominent consequence of the activation of the CWI pathway by cell wall stress is the induction of an adaptive transcriptional program coordinated by Slt2p/Mpk1p and mostly mediated by the transcription factor Rlm1p 28. Notably, we observed that deletion of *RLM1* led to resistance to acetic acid (Fig. 1B, C and D) and impaired acetic acid-induced cytochrome *c* release into the cytosol (Fig. 5). Furthermore, the *rlm1*Δ mutant was more susceptible to zymolyase digestion, in agreement with the phenotype of CWI mutants, described above. We therefore sought to determine the involvement of Rlm1p target genes in acetic acid-induced apoptosis. With the aid of bioinformatics tools, in particular the data available in the database YEASTRACT (http://www.yeastract.com ), we could identify 205 genes putatively regulated by Rlm1p, of which 29 are essential. To identify genes regulated by Rlm1p required for resistance to acetic acid induced-cell death, we screened the strains mutated in all the non-essential genes under Rlm1p control from the EUROSCARF haploid mutant deletion collection (EUROSCARF; http://web.uni-frankfurt.de/fb15/mikro/euroscarf ). The 176 mutant strains were patched onto 96-dot arrays and incubated in synthetic complete liquid medium (SC-Gal) containing 250 mM acetic acid at pH 3.0. The presence of viable cells was tested at 100, 200, 300 and 400 min and compared with that of wild type cells. Of the 176 mutants tested, 103 were more resistant to acetic acid-induced cell death and 28 were more sensitive while the other 45 mutants had a phenotype similar to that of the wild type strain (Table S1). To further validate our results, we determined the viability of 50 randomly selected mutant strains from the resistant and sensitive datasets and compared the phenotype with that obtained in the screening. The phenotype of 47 strains was confirmed, two mutant strains scored as sensitive in the 96-plate assay displayed no differences from wild type when tested individually, and one was more resistant in the 96-plate assay but also did not display any differences from wild type when tested individually (not shown).

In the dataset of resistant strains, the Biological Process most significantly enriched according to Gene Ontology classification (FUNSPEC analysis http://funspec.med.utoronto.ca was "fungal-type cell wall organization", enclosing genes coding for proteins involved in hydrolysis of O-glycosyl compounds (*EXG2*, *UTR2*, *CRH1*, *BGL2* and *EXG1*), namely glucan exo-1,3-beta-glucosidase activity (*EXG2*, *BGL2*, *EXG1*), cell wall proteins containing a putative GPI-attachment site (*PST1*, *YLR194C*), a putative GPI-anchored aspartic protease (*YPS6*), and cell wall mannoproteins (*CCW12*, *CCW14*) (Table 1). Deficiency in proteins Flc1p, Flc2p, Rim21p and Dfg5p, also involved in the "cell wall biogenesis", conferred resistance to acetic acid-induced cell death as well. These results indicate that cell wall remodeling plays a decisive role in the induction of apoptosis. Several genes with a function in polarized growth were also enriched, likely through their involvement in the modulation of Cdc42p and Rho proteins, essential for this process. These included *PXL1*, similar to metazoan paxillin, involved in adhesion, and *GIC2*, a Cdc42 effector, whose deletion conferred resistance to acetic acid. Accordingly, deletion of *BEM2,* a RhoGAP (Rho GTPase activating protein), resulted in sensitivity to acetic acid, presumably due to the increased Cdc42-GTP levels observed in this mutant 29.

**Table 1:** Categories that were significantly enriched (p-value below 0.01) based on physiological function of the genes whose deletion increases the resistance to acetic acid-induced cell death.

**Category, biological process**	**p-value**	**Genes in the dataset**
fungal-type cell wall organization [GO:0031505]	4.626e-07	*PST1 EXG2 UTR2 CRH1 BGL2 SLT2 SIM1 YPS6 CCW12 YLR194C EXG1 CCW14*
cellular cell wall organization [GO:0007047]	7.386e-06	*EXG2 UTR2 CRH1 BGL2 CCW12 YLR194C EXG1 DFG5 SUN4*
fungal-type cell wall biogenesis [GO:0009272]	1.567e-05	*FLC2 DFG5 RIM21 FLC1*
cell wall chitin metabolic process [GO:0006037]	0.0002317	*UTR2 CRH1*

In the data set of sensitive strains, the biological process most significantly enriched according to Gene Ontology classification was also "fungal-type cell wall organization", followed by "response to stress". The "response to stress" class included the cytosolic catalase (*CTT1*), the subunit of the threalose 6-phosphate synthase/phosphatase complex (*TLS1*), and a protein of unknown function (*HOR7*). The "fungal-type cell wall organization" class, in contrast with the genes represented in the dataset of resistant strains, was composed in this case of genes that code for proteins involved in the stability of the cell wall, namely O-mannosylated heat shock proteins (*HSP150* and *PIR3*) and cell wall manoproteins (*CWP1*, *CWP2*) (Table 2). Therefore, proteins regulated by Rlm1p that ensure the stability of the cell wall protect cells from acetic acid-induced cell death.

**Table 2:** Categories that were significantly enriched (p-value below 0.01) based on physiological function of the genes whose deletion increases the susceptibility to acetic acid-induced cell death.

**Category, biological process**	**p-value**	**Genes in the dataset**
fungal-type cell wall organization [GO:0031505]	0.001243	*HSP150 CWP1 CWP2 PIR3*
response to stress [GO:0006950]	0.002342	*CTT1 HSP150 TSL1 HOR7*

The phenotype of the *rlm1*Δ mutant is the result of several responses; since deletion of some Rlm1p target genes results in resistance (those involved in cell wall remodeling), and of others in sensitivity (those involved in cell wall stability), and the overall phenotype of the *rlm1*Δ mutant is resistance to acetic acid-induced cell death, the more prevalent Rlm1p-mediated response to acetic acid seems to be cell wall remodeling.

### DISCUSSION

In this study, we performed a comprehensive analysis of the MAPK signaling pathways involved in acetic acid-induced apoptotic cell death. Absence of Ste11p MAP kinase, shared by the mating-pheromone response, invasive growth/pseudohyphal development and HOG pathways, resulted in higher cell survival. However, since deficiency in the MAPK of the invasive growth/pseudohyphal development pathway, Kss1p, did not alter sensitivity to acetic acid, this pathway does not seem to be involved in apoptosis induced by acetic acid. On the other hand, although several components of the HOG signaling pathway, both specific and common to other MAPK pathways, have a pro-death role in this process, deletion of the MAPK of the HOG pathway tends to confer sensitivity to acetic acid. Therefore, the results support the interpretation that the HOG pathway does not play a relevant role in signaling acetic acid-induced apoptosis, or that it has a dual role. These results are in accordance with those obtained in a genome-wide screen for the identification of positive and negative regulators of acetic acid-induced cell death, where the HOG pathway was also not identified as relevant in this process 30. In this analysis, the term "Sporulation resulting in formation of a cellular spore" was enriched and, consistently, we found that the mating-pheromone response signals cell death. Indeed, deficiency in several components of this pathway, and particularly in its specific MAPK, resulted in higher resistance to acetic acid-induced apoptosis. Absence of different CWI pathway components also conferred resistance to acetic acid, sustaining that this pathway is another major mediator of acetic acid-induced apoptosis. Of the mutants in CWI sensors, only *mid2*Δ displayed a resistant phenotype, suggesting a possible role for this sensor. Other sensors may also play a role, as their function may be redundant, and deletion of multiple genes would be required. Since a crosstalk exists between MAPK pathways, we also cannot exclude activation by intracellular signals. Also, the CWI MAPK mutant *slt2*Δ was slightly less resistant to acetic acid-induced cell death than the other CWI mutants. This can reflect its involvement in other processes and differential regulation, such as by PTP genes, Knr4p, Cdc37p, or the Hsp90 chaperone 31,32,33. Our results also highlight the different involvement of MAPK pathways in resistance to acetic acid-induced cell death and to chronic exposure to acetic acid. Indeed, it has been previously shown that impairment of the HOG pathway results in increased sensitivity to growth in the presence of acetic acid, whereas deletion of *SLT2* had no effect 34 or resulted in reduced growth in acidic pH 35,36. Notably, both Slt2p and Hog1p were phosphorylated in response to sub-lethal concentrations of acetic acid 23,24, though we only observed the phosphorylation of Hog1p in response to lethal concentrations used in our assay, for the time points tested (not shown, and Figure S1). This shows that there is not always an obvious relation between protein phosphorylation and the response/phenotype of a particular pathway, as has been found in other studies (e.g., 33). In this study, we focused on how the CWI pathway regulates acetic acid-induced apoptosis.

The yeast cell wall is a strong and rigid barrier that protects cells from extreme changes in the environment. It has four major functions: 1) stabilization of internal osmotic conditions, 2) protection against physical stress, 3) maintenance of cell shape, which is a precondition for morphogenesis, and 4) a scaffold for proteins 37. It consists of an inner layer of load-bearing polysaccharides (glucan polymers and chitin), acting as a scaffold for a protective outer layer of mannoproteins that extend into the medium 37. The yeast cell wall is a dynamic structure, and its composition changes in response to several stress conditions, such as heat stress, hypo-osmotic shock, cell wall stress, as well as carbon source, nutrient, or oxygen availability and in the presence of acetic acid 38,39. Accordingly, exposure to acetic acid renders the cell wall more resistant to lyticase digestion, reflecting an adaptation mechanism that allows cells to grow better in the presence of this weak acid 27. Our results now show that, in contrast, a more resistant cell wall is not needed for higher resistance to acetic acid-induced cell death. Indeed, CWI mutants, known to display cell wall defects 26, were more sensitive to zymolyase digestion but more resistant to acetic acid-induced cell death. Therefore, in order to identify the relevant functions regulated by this MAPK pathway that are involved in the higher resistance to apoptosis induced by acetic acid, we screened for targets of Rlm1p, the main downstream mediator of Slt2p signaling.

Rlm1p targets comprise genes involved in a multitude of processes, which are not restricted to genes with a cell wall function. Accordingly, several classes were represented in the datasets of genes regulated by Rlm1p whose deletion resulted in altered sensitivity to acetic acid-induced cell death, including several previously implicated in this process. These classes include proteins involved in sphingolipid metabolism 40, as well as genes implicated in the oxidative stress response 41 and mitochondrial components 10,11,12,14,42. Modulation of the CWI pathway can therefore affect multiple functions involved in acetic acid-induced cell death. However, as expected, most genes found are involved in stabilization or remodeling of the cell wall, as well as vesicle trafficking and polarized growth, all affecting cell wall structure.

The results from our screen indicate that the stabilization of the cell wall is important for the cell's ability to resist to acetic acid-induced cell death, while cell's engagement in cell wall remodeling compromises its survival. Indeed, many genes required for cell wall stability were found in the sensitive dataset. Moreover, several genes involved in the modulation of Cdc42p and Rho proteins were found, which seem to be associated with the function of these proteins in polarized growth. Since polarized growth requires re-organization of the actin cytoskeleton as well as cell wall remodeling, these processes are intimately connected. This highlights the crosstalk between the CWI and mating-pheromone response MAPK pathways we found as mainly involved in acetic acid-induced cell death, and their intricate regulation.

The results obtained in this study may impact different biotechnological processes and biomedical applications. High levels of acetic acid produced during acid catalyzed-hydrolysis of lignocelluloses, used as raw material to produce bioethanol, or formed during industrial fermentation processes, often compromise the yeast fermentative performance 4,43. One way to overcome the inhibition of fermentation process is to render industrial strains more resistant to this weak acid. Identifying molecular determinants of sensitivity to acetic acid, and of strategies to increase strain resistance, is therefore of utmost importance. Specifically, modulation of upstream signaling pathways is of great interest, since a number of genes and processes are affected to produce a desirable outcome, rather than affecting specific downstream genes with limited functions, which the cells often adapt to through redundant/compensatory mechanisms. In the future, it will be interesting to determine how modulating the CWI signaling pathway impacts yeast fermentative performance, namely industrial ethanol production from lignocellulosic hydrolysates highly enriched in acetic acid.

Many of the cellular and metabolic features that constitute hallmarks of tumor cells include higher glycolytic energetic dependence, lower mitochondrial functionality, increased cell division and metabolite synthesis 44. Notably, these same alterations result in higher sensitivity of yeast cells to acetic acid 30, consistent with the specific sensitivity of CRC cells to short chain fatty acids, including acetate and propionate, and reinforcing the exploitation of yeast as a model system to elucidate the molecular basis of this sensitivity. Therefore, despite obvious differences between the extracellular matrix (ECM) and the yeast cell wall, it would be interesting to determine whether increased ECM dynamics could also underlie the higher susceptibility of CRC cells to acetate-induced apoptosis, or whether modulation of this process or of MAPK pathways could further potentiate the sensitivity of these cells to acetate, without compromising viability of healthy adjacent cells. Indeed, modulating MAPK signaling pathways has previously been suggested as a strategy in colorectal cancer treatment, though particular molecular components to be targeted have not been identified, nor has its efficacy been evaluated 45.

In summary, our work indicates that the mating-pheromone response and CWI MAPK pathways are involved in signaling acetic acid-induced cell death, as blocking signal transduction in these pathways renders cells more resistant to programmed cell death induced by acetic acid. This resistance is achieved through regulation of several processes, of which alterations in the cell wall were particularly evident. Modulation of the CWI MAPK signaling pathway therefore emerges as a powerful strategy to increase resistance of yeast strains to acetic acid through multiple effector processes, with potential application in biotechnology as a way to avoid stuck or sluggish alcoholic fermentations. Our results also open new avenues of research into the regulation of acetate-induced apoptosis in mammals, with particular impact for the design of novel therapeutic opportunities against colorectal carcinoma based on the modulation of MAPK pathways.

### MATERIALS AND METHODS

#### Yeast strains and growth conditions 

The yeast *S. cerevisiae* strain BY4741 (MATa *his3*Δ1 *leu2*Δ0 *met15*Δ0 *ura3*Δ0) 46 and isogenic mutant strains were used throughout this work, except for determination of the *BCK1-20* overexpression phenotype, where W303-1A was used due to auxotrophy requirements (*MATa*, *ura3-52*, *trp1Δ 2,* *leu2-3,112*, *his3-11*, *ade2-1*, *can1-100*). Cells were maintained in rich medium (YPD) (1% yeast extract, 2% glucose, 2% bacto-peptone, 2% agar) and grown in synthetic complete medium (SC-Gal) (0.67% Bacto-yeast nitrogen base w/o amino acids (Difco), 2% galactose and 0.2% Dropout mix). Galactose was used as the carbon and energy source to address mitochondrial function, as this leads to higher mitochondrial mass because galactose is less effective in the repression of respiratory metabolism 47. The sensitivity of several strains was assessed in YPD and the results were comparable (e.g., *rlm1*Δ, not shown).

#### Acetic acid treatments: quantitative c.f.u. counts

Yeast cells were grown overnight in liquid SC-Gal (or SC-Gal without tryptophan) until exponential growth-phase (OD_600nm_ = 0.5-0.6) at 30°C with agitation (200 rpm). Cells were harvested by centrifugation and suspended in fresh SC-Gal medium (pH 3.0) with 90-120 mM acetic acid, and incubated for 200 minutes at 30°C in 50 mL Erlenmeyer flasks with an air: liquid ratio of 5:1 in a mechanical shaker at 200 rpm. Samples were taken at different time points, diluted to 10^-4^ in 1:10 serial dilutions in deionized sterilized water, and 40 µL drops were spotted on YPD agar plates in replicates of seven. Colony forming units (c.f.u.) were counted after 48 h incubation at 30°C. Cell viability was calculated as percentage of c.f.u.s in relation to time zero.

#### Semi-quantitative spot assays:

Yeast cells were grown overnight in SC-Gal medium until exponential growth-phase (OD_600nm_ = 0.5-0.6) at 30°C at 200 rpm. Cells were harvested by centrifugation and suspended in fresh medium with 140 mM acetic acid, pH 3.0, 40 mM propionic acid, 120 mM butyric acid, 2 mM hydrogen peroxide, or 0.05% methyl methanesulfonate and incubated for 180 minutes at 30°C in 50 mL Erlenmeyer flasks with an air: liquid ratio of 5:1. Samples were taken at different time points, diluted to 10^-4^ in 1:10 serial dilutions in deionized sterilized water, and 5 µL drops of each dilution were spotted on YPD agar plates. Plates were photographed after incubation for 48 h at 30°C.

#### 96 well plate screen

Mutant strains deleted for Rlm1p target genes were patched in ordered arrays of 96 on YPD plates and grown at 30°C for 2 days. Yeast cells were inoculated into 96-well plates containing synthetic complete, 2% galactose medium with a pin-replicator, and grown for 24 hours at 30°C. Cultures were diluted 100 fold using a multichannel pipette into SC-Gal medium at pH 3.0, containing 250 mM acetic acid (this concentration was optimized for the culture conditions used in the 96 well plate screen). At different times of incubation (100, 200, 300 and 400 minutes), cells were replicated into 96-well plates containing YPD medium, using a pin replicator, as described in 30. After incubation at 30°C for two days, optical density (640 nm) was measured to assess cell growth reflecting the presence of viable cells in the inoculum, using a microplate reader (Molecular Devices SpectraMax Plus).

#### Zymolyase sensitivity assay 

To monitor structural changes in the yeast cell wall, a Zymolyase (Medac; Medacshop) sensitivity assay was performed as described in 48. Briefly, after treatment with 110 mM acetic acid for 200 min, cells were harvested by centrifugation, washed with sterile distilled water and resuspended in 0.1 mM sodium phosphate buffer (pH 7.5). After adding 60 µg/ml of Zymolyase, cell lysis was followed by measuring the decrease in the OD_600nm_ of each cell suspension.

#### Flow cytometry 

During acetic acid treatment, samples were also taken to assess loss of plasma membrane integrity and accumulation of reactive oxygen species (ROS) by flow cytometry, using an EPICS® XL™ (Beckman COULTER®) flow cytometer equipped with an argon-ion laser emitting a 488 nm beam at 15mW. Cells were collected by centrifugation, washed in deionized water, suspended in phosphate buffered saline (PBS) and stained with 1 µg/mL propidium iodide (PI, Sigma) or 2 µM/mL dihydroethidium (DHE, Sigma) for 10 and 30 min, respectively, at room temperature, in the dark. Monoparametric detection of PI fluorescence was performed using FL-3 (488/620 nm) and detection of DHE was performed using FL-4 (488/675 nm).

#### Assessment of cytochrome* c* release 

Mitochondrial and cytosolic fractions of untreated and acetic acid-treated cells were prepared as described in 14 and protein concentration determined using the Bradford method and BSA as standard 49. Mitochondrial integrity was assessed by measuring citrate synthase activity 14. Fractions were separated on a 12.5% SDS-polyacrylamide gel and transferred to a Hybond-P Polyvinylidene difluoride membrane (PVDF; GE Healthcare). Membranes were incubated with the primary antibodies mouse monoclonal anti-yeast phosphoglycerate kinase (Pgk1p) antibody (1:5000, Molecular Probes), mouse monoclonal anti-yeast porin (Por1p) antibody (1:10000, Molecular Probes) and rabbit polyclonal anti-yeast cytochrome *c* (Cyc1p) antibody (1:2000, custom-made by Millegen), followed by incubation with secondary antibodies against mouse or rabbit IgG-peroxidase (1:5000; Sigma Aldrich). Pgk1p and Por1p were used as a loading control for cytosolic and mitochondrial fractions, respectively. Immunodetection of bands was revealed by chemiluminescence (ECL, GE Healthcare).

### SUPPLEMENTAL MATERIAL

Click here for supplemental data file.

All supplemental data for this article are also available online at http://microbialcell.com/researcharticles/cell-wall-dynamics-modulate-acetic-acid-induced-apoptotic-cell-death-of-Saccharomyces-cerevisiae/.
