# Supplementary material for: Cell wall dynamics modulate acetic acid-induced apoptotic cell death of Saccharomyces cerevisiae
Source: Microb Cell. 2014 Aug 27;1(9):303–14. doi: 10.15698/mic2014.09.164 (PMC5349133; doi:10.15698/mic2014.09.164)
Supplement: Supplementary file 1 [file mic-01-303-s01.pdf]

Figure S1.

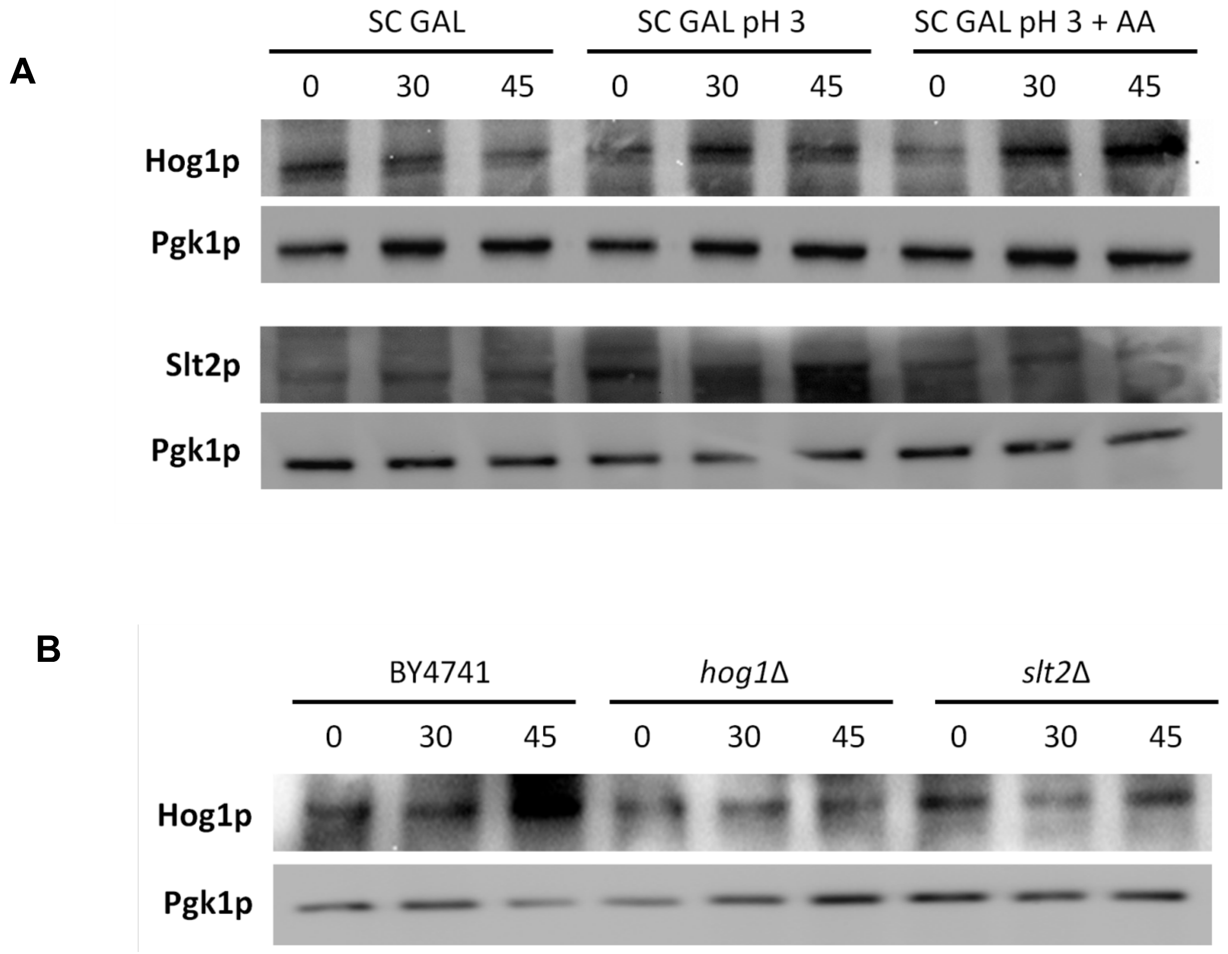

BY4741 cells (A) or cells of the indicated strains (B) were grown in SC Galactose to mid-exponential-phase (OD600 0.5-0.6), and then exposed to 120 mM of acetic acid (AA). At the indicated times, cells were harvested and processed, and the crude protein extracts were analyzed by Western blot. Proteins were separated by SDS-PAGE using 10% polyacrylamide gels and blotted onto a Hybond-P Polyvinylidene difluoride membrane (PVDF; GE Healthcare). The membrane was incubated with rabbit anti-phospho-p44/42 MAPK (Cell Signaling Technology, Beverly, MA, USA) at a 1:5000 dilution or rabbit anti-phospho-p38 MAPK (Cell Signaling Technology, Beverly, MA, USA) at a 1:50000 dilution as primary antibodies, to detect phospho-Slt2p, or phospho-Hog1p, respectively. For the detection of Pgk1p (loading control), mouse anti-Pgk1p (Sigma, St. Louis, MO, USA) at a 1:500 dilution was used as primary antibody. Subsequently, membranes were incubated with the secondary antibody, mouse and rabbit IgG-peroxidase (Sigma, St. Louis, MO, USA), at a 1:5000 dilution. Immunodetection was performed by chemiluminescence, using a kit from GE Healthcare.

Table S1

Genes whose deletion yielded a resistance phenotype
AFR1, AGE1, AGP3, AMN1, API2, ARI1, ASE1, ATG8, BGL2, BSC1, BUD22, CCW12, CCW14, CLB4, COY1, CRH1, DFG5, ECM19, ECM38, EXG1, EXG2, FAA4, FBP26, FIT2, FLC1, FLC2, FLO10, GDS1, GIC2, HSP12, ICS2, INO1, IPT1, IRC22, KDX1, KTR2, LHS1, LYS9, MAK32, MDM31, MGA1, MLP1, MSB4, MSC1, NFT1, NFU1, PRM5, PRY1, PRY2, PST1, PTP2, PXL1, RAD30, RAD55, RCK1, RIM21, RMD6, RNH203, RPA34, RPI1, RPL9b, RTS3, SBP1, SFG1, SGE1, SIM1, SLM5, SLT2, SNF11, SPS2, SRL3, SUN4, TAT2, UTR2, YBR071w, YCL049C, YDR417C, YEF1, YER001w , YGL159W, YGP1, YGR149W, YHL042W, YHR033W, YIL108W, YJL105w, YJL160c, YJL171C, YKE4, YKR104W, YLR040c, YLR194C, YMR103C, YMR295C, YMR315w, YNL058C, YNL208W, YOL159C, YOL160W, YPL088W, YPS5, YPS6.
**Genes whose deletion yielded a sensitive phenotype**
CHS3, CTT1, CWP1, CWP2, DSD1, FKS1, GVP36, HOG1, HOR7, HSP150, IME2, MCH5, MTF2, NNF2, PIR3, PUN1, RMD1, RML2, SMF1, SUS1, TPO2, TSL1, TUB3, VPS74, YDR042C, YSN1, YSP3, YSR3.
**Genes whose deletion yielded a phenotype similar to wild type**
ALD3, APQ12, ASK10, BER1, CHS1, CHS5, CIS3, CRG1, CSN9, DDR48, DSE2, EGD2, FMP33, FRE6, FRQ1, HAL1, MID2, MSC6, MUM2, PCL1, PIR1, POG1, PPR1, PRM10, SED1, SPO77, SPS100, SUR1, WSC4, YGL260W, YHL041W, YHR097C, YJL107C, YLR111W, YNL010W, YOR314W, YPR195C, YPS1, YPS3.
